# The Golden Retriever Lifetime Study: establishing an observational cohort study with translational relevance for human health

**DOI:** 10.1098/rstb.2014.0230

**Published:** 2015-07-19

**Authors:** Michael K. Guy, Rodney L. Page, Wayne A. Jensen, Patricia N. Olson, J. David Haworth, Erin E. Searfoss, Diane E. Brown

**Affiliations:** 1 Morris Animal Foundation, 720 S. Colorado Boulevard, Denver, CO 80246, USA; 2 Flint Animal Cancer Center, Colorado State University, Fort Collins, CO 80523, USA; 3 Department of Clinical Sciences, Colorado State University, Fort Collins, CO 80523, USA; 4 American Humane Association, 1808 Willow Springs Way, Fort Collins, CO 80528, USA

**Keywords:** canine, cancer, risk factors, prospective cohort, comparative medicine

## Abstract

The Golden Retriever Lifetime Study (GRLS) is the first prospective longitudinal study attempted in veterinary medicine to identify the major dietary, genetic and environmental risk factors for cancer and other important diseases in dogs. The GRLS is an observational study that will follow a cohort of 3000 purebred Golden Retrievers throughout their lives via annual online questionnaires from the dog owner and annual physical examinations and collection of biological samples by the primary care veterinarian. The field of comparative medicine investigating naturally occurring disorders in pets is specifically relevant to the many diseases that have a genetic basis for disease in both animals and humans, including cancer, blindness, metabolic and behavioural disorders and some neurodegenerative disorders. The opportunity for the GRLS to provide high-quality data for translational comparative medical initiatives in several disease categories is great. In particular, the opportunity to develop a lifetime dataset of lifestyle and activity, environmental exposure and diet history combined with simultaneous annual biological sample sets and detailed health outcomes will provide disease incidence data for this cohort of geographically dispersed dogs and associations with a wide variety of potential risk factors. The GRLS will provide a lifetime historical context, repeated biological sample sets and outcomes necessary to interrogate complex associations between genes and environmental influences and cancer.

## 1. The canine lifetime health project

Morris Animal Foundation, founded in 1948, is a public nonprofit organization that funds humane scientific studies to advance animal health. The Canine Lifetime Health Project (CLHP) was initiated by Morris Animal Foundation in 2012 as the first-of-its-kind database to register dog owners interested in participating in clinical research studies. The CLHP website (www.CanineLifetimeHealth.org) registers dog owners and their dogs of all ages and breeds including mixed-breed dogs. The concept of the CLHP website as the gateway to a community of dog owners accessible for separate health studies is similar to that used for human clinical research by the Army of Women (www.ArmyOfWomen.org; accessed 20 October 2014), where both women and men can register online to be eligible to participate in studies related to breast cancer. The Love/Avon Army of Women was started by the Dr. Susan Love Research Foundation in 2008 and has since registered over 375 000 people of which various subsets have participated in at least 19 studies.

The CLHP gathers basic demographic information from interested dog owners about themselves and their dog. Information about the dog's age, gender, breed and geographical location are included. As new study opportunities are presented, and then approved by the CLHP oversight team and Morris Animal Foundation scientific advisers, owners of study-eligible dogs are notified. Subsequent contact for such studies is coordinated directly by the proposed study's investigator. In addition to registering dog owners and their dogs, the CLHP also registers veterinarians who are interested in participating as study veterinarians for their clients and patients. Veterinarians register online at the same website (www.CanineLifetimeHealth.org) by providing their name, clinic name, address, telephone number and email address. As of 15 January 2015, there are 16 098 dogs registered in the CLHP database, including 15 601 purebred dogs comprising 32 breeds and 497 mixed-breed dogs. There are also 13 905 dog owners and 3621 veterinarians registered in the CLHP database. The CLHP, in addition to serving as a database of dog owners, dogs and veterinarians, also serves as an administrative umbrella under which clinical studies may be conducted by the Morris Animal Foundation. The Golden Retriever Lifetime Study (GRLS) is the first study to be initiated and conducted by Morris Animal Foundation under the auspices of the CLHP (see below).

## 2. The Golden Retriever Lifetime Study

The GRLS is structured on a concept created by the Framingham Heart Study (FHS) [1]), a long-term longitudinal study following a cohort of people to identify the important risk factors for heart disease that began in 1948. In 1972, the FHS recruited the children of the original participant cohort into a second cohort study, and in 2002 began following the third generation. The FHS is still contributing to scientific knowledge today with over 2500 separate publications of study results (www.FraminghamHeartStudy.org; accessed 20 October 2014). The GRLS is a prospective, observational, longitudinal cohort study of 3000 healthy Golden Retrievers enroled from six months to 2 years of age and followed throughout their lives in an effort to identify the incidence and important risk factors for cancer and other canine diseases. Participating owners and veterinarians provide informed consent prior to enrolment for purposes of biological sampling and medical record collections. A canine lifetime longitudinal cohort will provide a shorter time to the primary outcome of interest, cancer, compared with a human cohort study. The study anticipates reaching full enrolment of 3000 dogs by March 2015.

## 3. Value of prospective longitudinal studies

The GRLS is a prospective longitudinal cohort study. A cohort study is a defined group of individuals who are followed over a defined period of time [2]. Cohort studies form a suitable model to simultaneously evaluate both multiple exposures and multiple outcomes of disease [3]. Prospective cohort studies offer opportunity to collect data on time varying exposures and confounders. Golden Retrievers were selected as the canine population of interest for several reasons. They are a very popular breed in the USA which increases the likelihood of sufficient enrolment in a fiscally reasonable time. Following a purebred cohort of Golden Retrievers will help reduce the genetic variability that would be inherent in a mixed-breed dog population study. The Golden Retriever breed is also suspected of being at high risk for cancer development, as described below. Golden Retrievers are owned by a diverse population of humans which helps to create dispersed environmental exposures. The GRLS attempts to reduce selection bias, the selection of individuals for a study that are not representative of the larger population, by recruiting a large number of participants from all geographical regions throughout the contiguous United States, and by recruiting a diverse participant pool through broad outreach to dog owners, breeders and veterinarians.

## 4. Spontaneous cancers in dogs—the primary endpoint for the Golden Retriever Lifetime Study

Cancer is considered a leading cause of morbidity and mortality in pet dogs although the extent of the cancer burden on the health and longevity of dogs is not well characterized [4]. A formal cancer registry, no longer in use, was developed 60 years ago in the San Francisco area by the National Cancer Institute but since then there has not been a significantly accurate way to estimate changes in cancer incidence in the USA [5,6]. Current estimates of cancer frequency do exist from several large national databases such as the Veterinary Medical Database (VMDB) which represents a collection of medical record abstracts from most of the veterinary teaching hospitals associated with colleges of veterinary medicine in the USA. This dataset has been used for comparisons of canine cancer frequency by histological type, dog breed and other demographic characteristics but is inherently biased due to the population of dogs sampled at regional veterinary referral hospitals [7,8].

The absence of reporting standards for cancer in companion animals also provides a challenge. The definitive diagnosis of a suspected neoplastic condition is subject to cost considerations and access to the tissue for diagnostic purposes if it is not a superficial lesion. Cancer diagnoses reported from histopathological laboratory databases may reflect such reporting bias and underestimate the true frequency of intracavitary cancers within the thorax or abdomen such as splenic haemangiosarcoma, adrenal masses or primary pulmonary nodules owing to the need for an invasive biopsy technique which may be declined by owners at the time of clinical presentation.

One goal of the GRLS is to address some of the limitations of existing databases. The defined population of the GRLS (denominator) and the careful diagnosis of all benign and malignant conditions in this population (numerator), with financial support to dog owners for the annual examination as well as biopsy procedure, will permit a current and accurate calculation of incidence for some of the most commonly diagnosed and impactful cancers in this cohort of enroled Golden Retrievers, including lymphoma, osteosarcoma, haemangiosarcoma and mast cell tumour. The GRLS ensures the proper categorization of each diagnosis of cancer by a thorough review of each case by study scientists, the principal investigator and participating pathologists.

The aetiology of cancer is often a complex interaction between multiple driving genetic and/or epigenetic alterations which influence the generation of the neoplastic phenotype in the context of environmental influences that may modify the development of cancer directly or indirectly. Remarkable progress has been made in the last 10 years, since the sequencing of the canine genome, to characterize germ line or genomic DNA changes that account for cancer, and to begin to investigate the multifactorial, complex nature of somatic gene alterations that may be involved with cancer development in dogs. A recent review provides an interesting and salient assessment of this area [9]. It is pertinent to mention that studies reporting changes in chromosome or gene structures are conducted in sample sets of material collected at a single time point and very often without annotated medical record histories or endpoints. The GRLS will provide a lifetime historical context, repeated biological sample sets and outcomes necessary to interrogate complex associations between genes and environmental influences.

There have been few studies that attempt to identify nongenetic risk factors for cancer in dogs and this subject has been recently reviewed [4]. Such studies have most often been case–control observational studies conducted to survey specific cancers for statistically significant risk associations. An overview of the most significant risk factors currently known for cancer development in dogs include: reproductive hormone exposure and the development of mammary cancer [10], breed associations for several cancers including Scottish Terriers with bladder cancer [11] and Bernese Mountain dogs with histiocytic sarcoma [12], large or giant body size with osteosarcoma [13], and environmental exposures such as asbestos with mesothelioma and tobacco smoke exposure with lymphoma or nasal cancers in dolichocephalic dogs [14]. However, the level of evidence in veterinary medical epidemiology for such associations is otherwise generally too weak to draw solid conclusions. For example, a recent systematic review concluded that the association between reproductive hormone presence and mammary cancer in dogs is not sufficiently robust, based on the rigorous requirements of a formal systematic review, to conclude a direct association [15]. This conclusion may be a reflection of the poor level of evidence available for such formal analyses in this area and well planned, high-evidence value clinical studies designed to provide the data sufficient to make valid conclusions are needed in veterinary medicine. It is also clear that environmental modification of genetically confirmed cancers may accentuate or ameliorate the expression of such cancers. For instance, a diet consisting of vegetables such as carrots was demonstrated to reduce the risk of bladder cancer development in Scottish Terriers [16], whereas herbicide and insecticide exposures were reported to increase the risk [17].

Diet and energy balance have been intensively studied as cancer risk factors in humans. It is notable that obesity has not been identified as having influence on the development of cancer in dogs, and neither dogs fed a high fat diet nor obese dogs had a higher rate of mammary cancer compared with non-obese dogs [18]. With the collection of annual body condition scores, the GRLS may be particularly useful for quantifying the risk of obesity on cancer occurrence as well as many other conditions where obesity has been associated with increased risk for disease. Likewise, other dietary issues such as hypovitaminosis D and supplementation of omega-3 fatty acids on the expression of sensitive cancers may be especially well examined in a prospective lifetime cohort study such as the GRLS.

Several areas of current investigation related to cancer aetiology include the role of chronic inflammation, epigenetic modulation of gene expression and metagenomics (the vast numbers of microorganisms that populate specific niches of the body such as the gastrointestinal system, oral cavity or skin). Thorough archival collections, such as those generated by the GRLS, will be required to develop appropriate hypotheses as to how these factors may be manifested in clinical syndromes such as cancer.

The anticipated frequency of cancer in this cohort of Golden Retrievers is estimated to be approximately 60% based upon a Golden Retriever Club of America survey (http://www.grca.org/pdf/health/healthsurvey.pdf; accessed 4 February 2015). The lack of accurate incidence data for cancer is one of the main problems in veterinary medicine. The primary study objective is to document an estimated 500 of the most impactful cancers in Golden Retrievers during the study period. Table 1 identifies the primary and secondary endpoints of the GRLS.

**Table 1.** Primary and secondary endpoints for the GRLS.

primary endpoints	secondary endpoints
determine the incidence of four significant types of canine cancer (haemangiosarcoma, osteosarcoma, lymphoma and mast cell tumour) in this cohort of Golden Retrievers	estimate the incidence and risk factors for common health disorders other than cancer
identify germline genetic variants associated with common cancers	explore associations between genetic variations, potential health, dietary and environmental risk factors and the development of other specific cancers
characterize the lifestyle, environmental, reproductive and nutritional risk factors associated with cancer development of four significant types of cancer	
establish extensive data and biological sample repositories for future analyses of major diseases, disorders or conditions in Golden Retrievers	

## 5. Material and methods

### (a) Structure of the Golden Retriever Lifetime Study

Following a purebred cohort of Golden Retrievers that includes the three-generation pedigrees and collection of DNA samples from study dogs will permit detailed genetic studies with a goal of identifying specific genes that may be correlated with certain forms of cancer. Given 500 expected tumours at 10 years, hazard ratios (HR) as small as 1.3 (e.g. 77.4% versus 82.6% rate of not developing cancer) can be detected with 90% power, at the 0.05 alpha level (two-sided), and only 375 tumours would be required to achieve 80% power. Larger HR, say 2% (e.g. 74% versus 86% rate of not developing cancer), obtain 90% power with approximately 100 cancer events. Similarly, less common cancers also lead to larger HR, so fewer cancer events are required.

Because both dog owners and veterinarians are required participants in the GRLS, separate recruitment efforts were directed toward each group. Dog owners (and breeders) were recruited through the Morris Animal Foundation website. The Golden Retriever Club of America (GRCA) offered a link to the study on their website and advertising space in their magazine. GRCA also provided a booth at national and regional Golden Retriever specialty competitions where Morris Animal Foundation staff could meet and speak with prospective study participants. The American Kennel Club (AKC) and the United Kennel Club (UKC) both provided outreach to their members. Additional advertising was also done in other magazines directed to pet owners.

A one-page study brochure was created that described the study and the requirements for both dog owners and veterinarians. These brochures were made available upon request at the study website. Foundation staff also created a volunteer network that used Facebook and other social media to raise awareness of the study among Golden Retriever owners and breeders, veterinarians and volunteers. A grassroots volunteer network quickly arose to tell others about the study and distribute brochures at local events. This volunteer network was one of the primary contributors to successfully increasing enrolment rates. Outreach to veterinarians was additionally accomplished through advertising in veterinary professional journals and through speaking engagements and vendor booths at national veterinary conferences where Foundation staff were able to also meet with prospective study veterinarians. The volunteer network was also helpful in raising study awareness among veterinarians.

Enrolment into the GRLS is an owner-driven process that begins when the owner of a study-eligible Golden Retriever accesses the CLHP website (www.CanineLifetimeHealth.org) and registers their dog. The system notes that the owner has a purebred Golden Retriever less than two years of age and living within the contiguous 48 United States, and then sends an email inviting the owner to apply for the GRLS. Formal inclusion and exclusion criteria are detailed in table 2.

**Table 2.** Inclusion and exclusion criteria for the GRLS.

owner inclusion criteria
— must be 18 years of age or older, reside in the contiguous USA, be willing to provide registration and identification information for enroled dog and dam and sire
— permit study team to access appropriate registry to retrieve full pedigree
— complete online questionnaires successfully prior to enrolment
— sign informed consent to participate with study requirements including annual veterinary visits, laboratory evaluations and sample procurement for biorepository storage
— consent to have samples of tumour tissue and normal tissue sampled and stored at the time of biopsy or surgery
— consent to relinquish rights to biological samples from their pet
— consider necropsy (post-mortem) evaluation at the time of death
owner exclusion criteria
— inability to complete pre-enrolment online survey/questionnaire successfully
Golden Retriever inclusion criteria
— American Kennel Club, United Kennel Club, other kennel club registration or service dog organization registration as a Golden Retriever with three generations of pedigree documentation
— less than two years of age at the time of study application
— no more than two littermates may be enroled from the same household
— microchip in place or owner willing to allow microchip or alternate permanent identification such as a tattoo
Golden Retriever exclusion criteria
— prior malignancy (benign lipomas or papillomas are allowed)
— prior diagnosis with a life-threatening condition that may substantially shorten expected lifespan
— inability to demonstrate a verified three-generation pedigree
veterinarian inclusion criteria
— licenced in the contiguous United States to practice in their State and in good standing
— agree to care for Golden Retriever enroled in the GRLS including commitment to complete study questionnaires online, biological sample collections and physical examinations
— provide medical records at the request of the owner to the owner or the GRLS study team
— perform and document diagnostic sample collection procedures, as appropriate, to confirm a diagnosis of cancer or refer dog to a specialty centre for collection of tumour tissue
— participate with the GRLS Team regarding education of client and hospital staff about the importance of this study to future canine health by providing counselling, in-service training and dissemination of GRLS communications

The application process begins with two one-time events: the first is completion of the Owner Profile with basic demographic information, and the second is an Owner Consent Form that the owner must read and sign electronically. The consent form states, among other things, that the owner agrees to participate in the study for the life of their dog through completion of an annual online health and lifestyle questionnaire that includes queries about the dog's dam and sire, and that they will see their veterinarian annually for their dog's physical examination, review of medical history and collection of biological samples (blood, urine, faeces, hair and nail trimmings). Once the Owner Profile and Consent Form are complete, the baseline Owner Questionnaire is made available to the applicant owner for completion online. A partial list of the contents of the Owner Questionnaire is shown in table 3.

**Table 3.** Categories of survey questions included in the owner and veterinarian baseline surveys used in the GRLS.

owner baseline questionnaire
— owner's primary and secondary home address and contact information
— dog's pedigree and registration information on dog, dam and sire (if known)
— primary and secondary lifestyle (pet/companion, obedience, agility, service, breeder, etc.)
— travel history
— reproductive history
— physical activity (table provided to record types of activities)
— use of over-the-counter (OTC) medications
— use of products for flea and tick control and for heartworm prevention
— history and practice of dental hygiene at home
— grooming history and use of types of products
— diet and feeding practices
— environment, living conditions and exposures
— 100-question behavioural questionnaire (C-BARQ)
veterinary baseline questionnaire
— confirm age and gender of patient
— confirm microchip or tattoo
— indicate whether dog was fasting at time of examination and sample collection
— review the medical history of patient
— review the medical history of patient's dam
— review the medical history of patient's sire
— general findings from the physical examination including weight, height at withers and estimate of Purina Body Fat Index (BFI)
— identification of any abnormalities by 15 different organ systems
— identification of superficial masses by location and size, includes dermal map
— any current or previous medications
— any current or previous vaccines

Within the baseline Owner Questionnaire, the owner provides the breed registration information for their dog (American Kennel Club, United Kennel Club or other registration information) as well as the registration information for the dog's dam and sire (if known). The CLHP study team uses this registration information to verify each pedigree back three generations, and this verification of the pedigree information is required before a dog can become enroled.

After the Owner Questionnaire is completed, owners are directed to schedule their baseline screening examination with their veterinarian. A sample collection kit is shipped to each owner with instructions to take the kit to their veterinarian a few days before the study examination. Veterinarians must register their clinic or hospital in the CLHP database in order to participate in the study. At the screening examination, the veterinarian will review the dog's history and the history of the dam and sire (if known by the owner; see table 3), assess the body condition score, conduct a physical examination to determine the health status of the Golden Retriever and acquire samples for routine laboratory tests (serum biochemistry profile, complete blood count (CBC), urinalysis, faecal examination for ova and parasites, heartworm antigen test and thyroid hormone (Total T4) level). Samples are also submitted to a biorepository for long-term storage (serum, urine, whole blood for DNA, hair, toe nails and faeces). See table 4 for details on sample collection and storage. The veterinarian or staff will complete the electronic veterinary survey and data capture forms (table 3). The veterinarian is officially responsible for confirming the health status of each enrolee according to inclusion and exclusion criteria (table 2) for the formal enrolment decision to proceed.

**Table 4.** Sample types, sample volumes, purpose of samples and long-term storage conditions for samples collected annually during the Golden Retriever Lifetime Study.

sample type	volume collected	purpose	storage conditions
for bio-bank storage			
whole blood—year 1 only	10 ml	extracted to DNA for bio-bank storage	−80°C
whole blood—all other years	10 ml	bio-bank storage of 10 × 1 ml whole blood aliquots	−80°C
serum	10 ml	bio-bank storage of 10 × 1 ml serum aliquots	−196°C
urine	5 ml	bio-bank storage of 5 × 1 ml urine aliquots	−80°C
hair	¼′ diameter, clipped	bio-bank storage of hair trimmings in one vial	−80°C
nails	5–10 nail trimmings	bio-bank storage of nail trimmings in one vial	−80°C
faeces	1 g	bio-bank storage of faeces in one vial	−80°C
tissues	<1 g	bio-bank storage of tissues in RNA*later*	−80°C
for laboratory use			
whole blood	3 ml	CBC	
serum	3 ml	chemistry profile, Total T4, heartworm antigen	
urine	5 ml	urinalysis	
faeces	1 g	faecal analysis for ova and parasites	
tissues	varies	histopathology analysis	

Veterinarians are not paid for their study participation by Morris Animal Foundation. They do receive a full chemistry, haematology, faecal and urinalysis panel on their patient annually at no charge. In addition, any potentially malignant histopathology specimens submitted by the veterinarian will be read by two veterinary pathologists and the results provided at no charge to the veterinarian. Finally, veterinarians have the opportunity to build a long-lasting relationship with a dedicated client, to be listed on a registry indicating their participation as a GRLS study veterinarian, and to participate in a groundbreaking research study. The study database was established with the idea that, during a 12–14 year study, owners or veterinarians may move and veterinarians will retire. Within the online system is a searchable feature for owners to locate veterinarians already participating in GRLS. If an owner cannot find another local veterinarian in the database, the study has the ability to help train new veterinarians for study participation.

Once formal enrolment has occurred, annual owner's responsibilities throughout the study will include completion of annual online questionnaires, notifying the system whenever a health event requires a visit to their veterinarian between annual veterinary visits, completion of a 4 day diet diary, and an annual veterinary visit. Sample collection kits and instructions are mailed annually to the owner prior to the veterinary visit for sample acquisition.

Annual visits to the owner's veterinarian are required for all Golden Retrievers enroled in the GRLS and include a medical history and physical examination, veterinary questionnaire of the dog's health and disease status by body system, and sample collections (described above) for both routine laboratory tests and sample collection for long-term biorepository storage. The information obtained from annual visits are summarized in the standardized forms provided through the electronic data capture systems. The annual comprehensive diagnostic panel is the same as the screening profile and includes a serum biochemistry profile, CBC, urinalysis, faecal analysis for ova and parasites, heartworm antigen and Total T4, and the results are distributed to the veterinarian annually for sharing with the owner.

Medical concerns about the health of dogs may occur between annual veterinary visits and may increase with the age of the dog. Many of these concerns may not require a call or visit to their veterinarian but are managed with appropriate at-home interventions such as first aid, rest and over-the-counter symptom control that do not require contact with the veterinarian or the GRLS study staff. Owners are asked to document these events for later inclusion in the annual questionnaire. When a health concern does require a call to the veterinarian and results in a veterinary visit, the owner will notify the GRLS study staff via the electronic data capture system of the date the veterinary visit occurred. If a study dog should go to a referral specialist or an emergency clinic, the regular veterinarian will be notified and is responsible for entering this information into the database once these records are shared with the regular veterinarian. When necessary, the study will communicate directly with these referral or emergency clinics to gather the appropriate records.

Veterinary visits will be accurately documented including history, physical examination, laboratory test results, assessment of concerns and plans for follow-up as well as medications dispensed, recommended and prescribed, and any other diagnostic procedures performed. Information regarding such visits will be managed using the electronic data capture system. The medical management of enroled Golden Retrievers will be coded using a systemized process for future data searches and information retrieval. During a veterinary visit, if a cancer is suspected, the veterinarian will determine the need for diagnostic and staging procedures to adequately manage the tumour. This may include laboratory screening tests, imaging and/or a fine needle aspirate or biopsy, if appropriate. Any suspected neoplastic condition is reported to inform the GRLS team of planned biopsy or sample procurement procedures. Because tumour incidence is an essential endpoint of the study, specific biopsy protocols and biopsy kits are provided to study veterinarians, along with procedures to be followed for sample collection, processing and diagnostics. Histopathological analysis of biopsy tissues is provided to participating veterinarians at no charge. Tumour tissues are processed for full histopathological assessment by board-certified veterinary pathologists and include a routine second opinion by a veterinary pathologist, and the tissue blocks and any prepared slides are archived. Tumour and adjacent normal tissue are also requested and processed at the time of biopsy or tumour removal to allow for future recovery of DNA and RNA. Diagnoses, whether related to cancer or other disorders, are subject to adjudication and confirmation by a separate Adjudication Committee of ad hoc specialists as directed by Morris Animal Foundation.

Owners of Golden Retrievers enroled in the GRLS are informed of the importance of full evaluation at the time of their dog's death and are highly encouraged from the beginning of their association with the study to have a necropsy performed. A necropsy kit for participating veterinarians is provided free of charge. Histopathological analysis of all necropsy tissues is also provided to participating veterinarians at no charge. Tumour samples are collected, analysed and archived as defined above for biopsy samples. The long-term storage for all samples is anticipated to be at least 10 years, with plans for continuous and active internal and external analyses by investigators with approved study protocols.

As sample sets are received at each laboratory, a daily inventory of new samples is created and the study is immediately notified if any samples are short or missing. This allows the study to request the owner and veterinarian to collect and ship additional samples from the dog. Dedicated staff audit the laboratory and questionnaire datasets for completeness, to ensure that participants are entering the information properly and that any missing data points are identified. Free text responses are reviewed and coded appropriately.

### (b) Study oversight

There are several levels of oversight for projects managed by Morris Animal Foundation under CLHP. An Oversight Committee (OC) consisting of Morris Animal Foundation Trustees ensures that the operational details of all studies, including the GRLS, are performed within the guidelines of the foundation's mission, and the study objectives and budgets are approved by the Morris Animal Foundation Board of Trustees. The OC ensures that the scientific, business and fiduciary objectives of the project are being met and that the scientific quality and rigour of the studies can withstand independent review.

A Scientific Steering Committee (SSC) of independent experts convened by Morris Animal Foundation provides expertise, advice and guidance about the planning and conduct of all studies, including the GRLS, and provides independent oversight to ensure the highest standards of animal welfare are met. The SSC consists of experts in diverse areas of biomedical science including toxicology, genetics, nutrition, cancer biology, oncology, behaviour and veterinary and human epidemiology, as well as members of the Golden Retriever Club of America leadership. Representatives from corporate partners are also members of the extended SSC. Current platinum corporate partners include The Mark and Bette Morris Family Foundation, Antech Diagnostics (VCA, Inc.), Zoetis, Inc., The Blue Buffalo Foundation for Cancer Research and Petco Foundation. Other partners include The Golden Retriever Foundation, Mars Veterinary and the Hadley and Marion Stuart Foundation. The SSC provides guidance on the study design and execution and provides expertise on issues related to data capture, sample collection and storage, and sample quality assurance standards. Since a comprehensive longitudinal lifetime study of this nature is unique to animal health, leveraging the experience and expertise of scientists from human health is important for the GRLS to withstand the highest levels of independent scientific scrutiny.

### (c) Study progress

**Figure 1.**
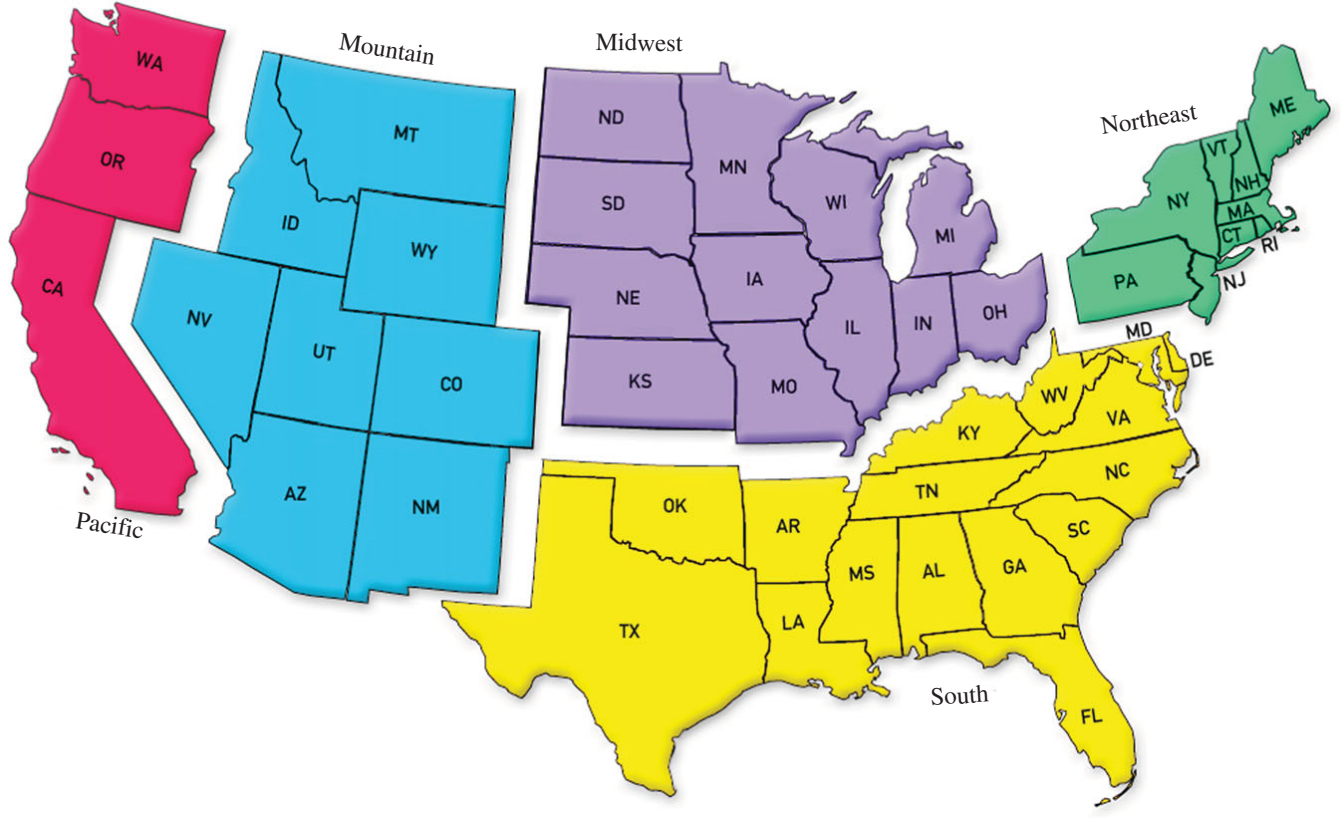
Geographical enrolment regions for the GRLS.

**Figure 2.**
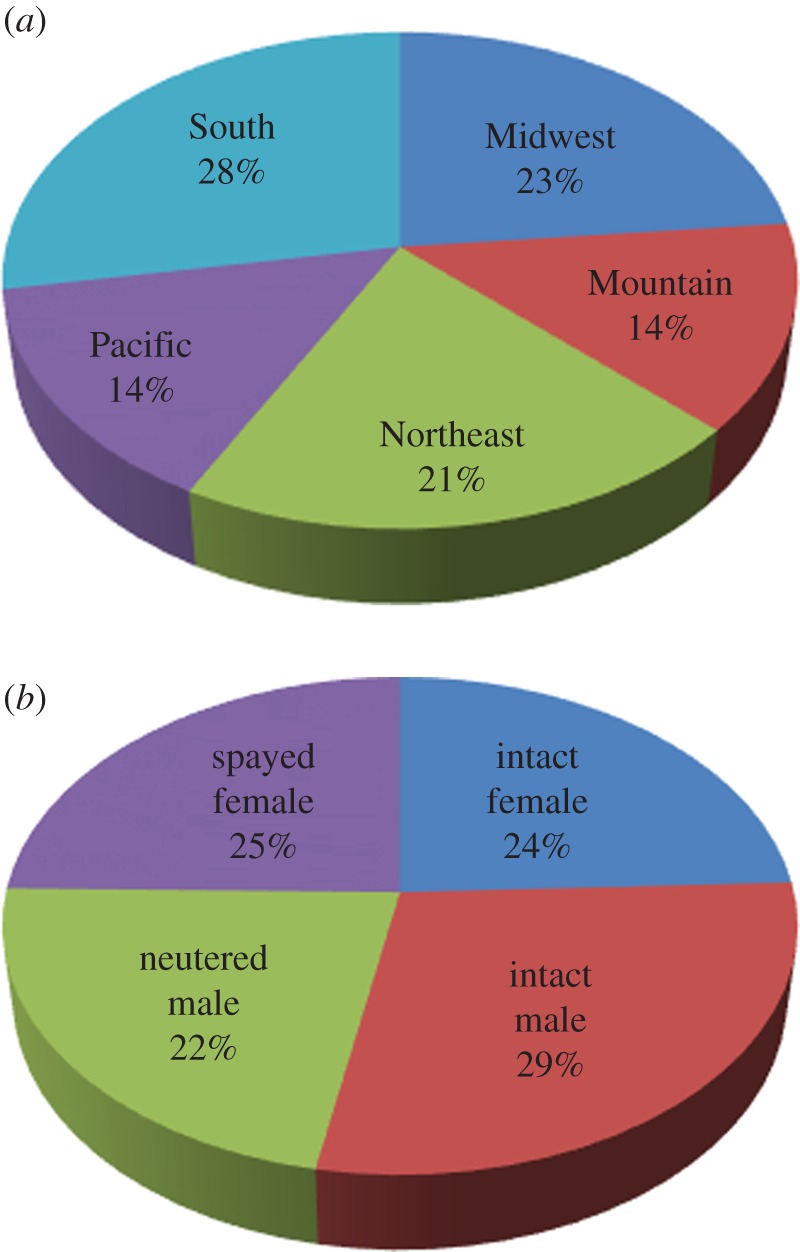
Distribution of 2550 enroled dogs in the GRLS as a function of US geographical region (*a*) and gender category (*b*) as of 15 January 2015.

The goal for the GRLS is to enrol 3000 purebred Golden Retrievers under 2 years of age. One enrolment goal is to enrol 20% of the study dogs in each of five geographical regions of the contiguous United States (figure 1). Another enrolment goal is to enrol half female dogs and half male dogs, and further within each gender to have half reproductively intact dogs and half neutered or spayed, for an overall goal of 25% of dogs in each of the four reproductive status categories at the time of enrolment. To date (15 January 2015), there are 2550 Golden Retrievers enroled in the GRLS, and enrolment is balanced by both geographical and gender distributions (figure 2). The study anticipates reaching the full enrolment of 3000 dogs by March 2015.

### (d) Translational opportunities of Golden Retriever Lifetime Study to human health

Naturally occurring diseases and conditions in companion animals have been recognized as valuable correlates of disorders that affect humans in both the biology of disease development and for purposes of early detection and treatment. The field of comparative medicine investigating naturally occurring disorders in pets is specifically relevant to the many diseases that have a genetic basis for disease in both animals and humans, including cancer, blindness, metabolic and behavioural disorders and some neurodegenerative disorders. The opportunity of the GRLS to provide high-quality data for translational comparative medical initiatives in several disease categories is great. In particular, the opportunity to develop a lifetime dataset of lifestyle and activity, environmental exposure and diet history combined with simultaneous annual biological sample sets will facilitate association studies between important shared health outcomes and a wide variety of potential risk factors. Such analyses might expedite the development or modification of future human health investigations. Importantly, the entire data and sample accumulation phase for the GRLS will be complete in 10–14 years and will include the opportunity to expand this cohort to numerous other parallel studies including the evaluation of multi-generational issues and sibling associations. Comparable work in humans requires decades.

The fundamental biology of disease development, progression and clinical expression is remarkably similar between humans and dogs for many conditions. This is the current motivation for the concept of ‘One Health’ and has received significant emphasis across disciplines as a means to accelerate and more efficiently address major medical issues. Infectious diseases, orthopaedic degenerative disorders and cancers have been most often identified as commonly affecting humans and animals in substantial frequencies. In addition, significant health issues in humans result from such modifiable risk factors as obesity and environmental tobacco smoke. GRLS has been constructed to address many of the same pre-disposing conditions that may affect humans and to provide the biological materials to address specific hypotheses. For instance, the role of chronic inflammation in cancer development may be considered in the GRLS population as can the influence of diet and microbiome on gastrointestinal function and dysfunction. Dogs are among only a few species besides humans that naturally develop autoimmune diseases with many similarities in the demographics and clinical expression of the spectrum of these disorders. The GRLS is an observational study and as such specific interventional studies are not anticipated. However, nested studies or parallel interventional studies are anticipated to be valuable extensions to the GRLS and provide specific opportunities. Interestingly, the general concept of ageing biology might be advanced substantially by using the GRLS resources as a means to define and validate functional ageing parameters in disorders such as cardiac, renal and neurologic performance as a function of age.

Personalized (precision) medicine is an emerging field where characterization of an individual's metabolic capacity to manage external or internal exposure to numerous compounds has much to gain from studies in companion animals. Dogs have similar phase I and phase II enzyme systems as humans which may be used to better understand drug toxicity/efficacy and drug-drug interactions that would benefit both species. Data on the multitude of medications that are administered over the lifetime of a Golden Retriever, many of which are products marketed for human use, are collected with the GRLS dataset along with clinical assessment of benefit or toxicity. Coupling this data with an analysis of pharmacogenomics data from the bio-sample set may have mutual benefits for dogs and humans.

Pets are also partners in our shared environment and are exposed in both similar and different ways that have potentially useful implications for human health. Some examples of comparable conditions resulting from indoor exposures include thyroid dysfunction resulting from flame retardant exposures in cats [19], asbestos and mesothelioma development in humans and dogs in the same household [20], indoor environmental air quality and respiratory disease or cancer in dogs, cats and humans [21]. Outdoor exposures are also of mutual interest and concern to pet and human health. The variety of activities of dogs in the GRLS parallels that of their owners with diverse opportunities to share environmental exposures. Dogs are often even more highly exposed due to intimate skin and oral exposures, accumulation of materials on hair that is subsequently ingested through self-grooming and a greater frequency of exposures to ground water and soil contaminants. Co-exposures to herbicides/insecticides from lawn, garden or community spaces for urban and suburban dogs or an agricultural-based exposure for rural dogs is also of importance to their human family. There are several studies examining exposure levels of such products in dogs [22,23]. The GRLS population is well suited to address such regional and lifestyle exposures as dogs are enroled from all regions of the contiguous United States where differing levels of exposures to distinct products are likely.

Dogs are omnivores, as are humans, which may be of relevance to general dietary components and potential exposure concerns. The majority of dogs in the GRLS not only consume a commercial dog food but also consume a great variety of vegetables, fruits, proteins and supplements. These details of dietary intake are being collected. As already mentioned, Scottish Terriers ingesting vegetables frequently had reduced risk of bladder cancer development [16]. A recent report identified high concentrations of PhIP (2-amino-1-methyl-6-phehylimidazo [4,5-b] pyridine) accumulating in the hair of dogs consuming cooked meat, also suggesting some opportunities to examine risks of outcomes of concern to humans [24]. Dogs are unable to synthesize vitamin D precursors from sunlight and must consume vitamin D in the diet. Hypovitaminosis D has been described as a risk factor for some diseases in dogs [25,26] and is also of significant interest to human health.

The collective GRLS resources offer a substantial opportunity to human and veterinary health professionals. A study involving similar data and samples acquired from owners of dogs enroled in the GRLS create a powerful example of ‘One Health’. In order to truly realize the opportunities for shared health investigations, funding to extend the GRLS dataset to other ancillary studies will be needed and will require engagement of public health investigators. The lessons learned from the establishment and execution of the GRLS may be one of the most valuable resources for such future research.

## 6. Relevance to Peto's paradox

The development of cancer in the canine species provides several interesting observations relative to Peto's paradox [27]. The premise of the paradox is based upon evidence that the frequency of cancer development is independent of the quantity of cells within an organism when considered across the spectrum of all mammalian species. The canine species has the most conformational diversity of all terrestrial mammals. Within the canine species, it has been observed that large breed dogs have a greater frequency of cancer development than do small dogs [7]. Thus, Peto's paradox does not seemingly apply to the canine species. What might be some of the reasons that dogs appear to represent an inconsistency with this theory?

The genetic influence of size variation on dogs has been linked to haplotype differences in the insulin-like growth factor 1 gene (IGF1) [28]. However, it is currently unknown if IGF1/IGF1-receptor biology also influences cancer frequency between large and small breeds, although it is tempting to speculate that cancer frequency may be linked to genetic determinants of size.

The plasticity of the canine genome that was exploited for purpose breeding, irrespective of size, over a relatively short time frame may have also resulted in variable cancer frequency between breeds. Working and sporting breeds such as the Bernese Mountain Dog, the Flat-coated Retriever and the Golden Retriever are considered to be at high risk of certain cancers, whereas small and toy breeds are at relatively low risk of cancer with one notable exception: the Scottish Terrier is 17× more likely to develop bladder cancer than other breeds [29].

The longevity of domestic dogs in the twenty-first century varies considerably from wild/feral dogs or even domestic dogs living in the latter half of the twentieth century. Whereas trauma, secondary infection or reproductive failure may be the leading causes of short lifespans or death in wild dogs [30], domestic dogs, and particularly dogs that are well cared for as members of the human family, with access to more sophisticated preventive and medical care, have an extended lifespan and survive to be afflicted more often by ‘old age’ disorders such as cancer. Thus, comparisons between wild and domestic canid species may not be valid.

It is well known that the major types of cancers that impact the lives of dogs (lymphoma, osteosarcoma and other sarcomas) differ from the leading causes of cancer deaths in people (lung, breast, colon and prostate; http://www.cancer.org/research/cancerfactsstatistics/cancerfactsfigures2014/; accessed 4 February 2015), which may suggest that the process of carcinogenesis between species is significantly influenced by differing gene–environment interactions or epigenetic modifications to such an extent that the patterns of cancer may not be comparable (e.g. tobacco smoke and lung cancer in humans, the role of obesity in cancer aetiology in humans and the absence of such evidence in dogs, oestrogen ablation in female dogs spayed before puberty and the resulting low breast cancer burden).

It is possible that one or more of these factors may effectively result in a conflict of Peto's paradox for the canine species, and several of these questions may be answered by the GRLS.

## Note added in proof

The study cohort of Golden Retrievers was fully enrolled (3000 dogs) as of March 24, 2015.
